# Direct Laser Writing of Functional Material with Independently Tunable Electrical and Mechanical Properties

**DOI:** 10.1021/acsomega.6c04927

**Published:** 2026-09-28

**Authors:** Yifan Chen, Wing Ki Lo, Zeying Chen, Sen Yang

**Affiliations:** Department of Physics, 58207 The Hong Kong University of Science and Technology, Clear Water Bay, Kowloon, Hong Kong 999077, China

## Abstract

Functional materials with tunable electrical and mechanical properties are essential for technologies such as flexible electronics and soft robotics, where the device performance must adapt to complex mechanical and electrical environments. Conventional approaches often rely on blending conductive metal nanoparticles (NPs) into a polymer matrix, where the pursuit of high conductivity can compromise mechanical integrity, and vice versa. We report a reagent-engineering strategy for direct laser writing (DLW) that decouples this fabrication challenge. By formulating a precursor that combines a metal salt or metalate with a suspension of NPs as a reducing agent, we achieve the single-step deposition of homogeneous metal-NP composites. Electrical characterization reveals that conductivity is primarily governed by the choice of metal salt or metalate, spanning several orders of magnitude, while changes in the NP filler induce only a moderate variation. Mechanical nanoindentation shows that both the elastic modulus and the hardness of the composite are controlled by the size and type of the NP suspension, indicating its potential role as a mechanical reinforcement phase. Energy-dispersive X-ray spectroscopy (EDS) confirmed a homogeneous distribution of both controlling phases within the deposit. This work demonstrates a direct-write platform where electrical and mechanical properties can be independently tuned through precursor design, offering a route to functionally graded materials.

## Graphical abstract

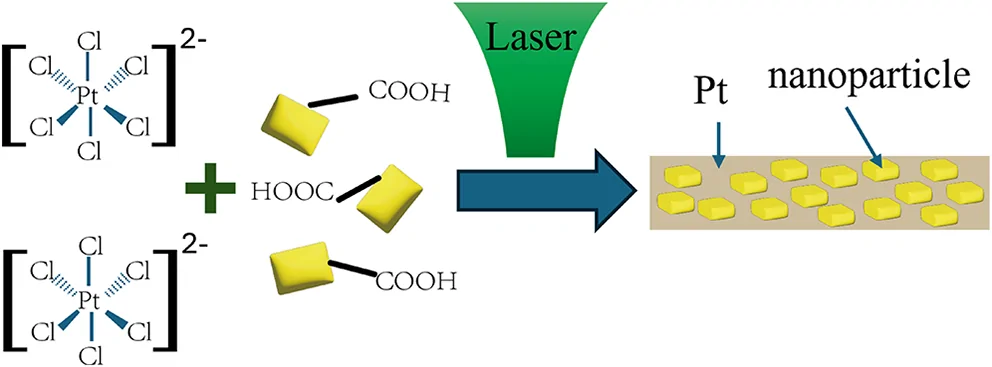

## Introduction

The development of materials with spatially programmable electrical and mechanical properties is a cornerstone for next-generation microsystems,^1,2^ soft electronics,^3–5^ and biointegrated electronics.^6–8^ These applications require devices that can seamlessly interface with dynamic environments,^9^ where localized regions may demand high conductivity, specific stiffness, or a unique combination thereof. The ability to tune these properties independently and integrate them within a monolithic structure is therefore not merely advantageous, but essential for sophisticated functionality.

Conventional strategies for fabricating such functional composites typically involve blending conductive fillers, such as metal nanoparticles (NPs), into a stretchable polymer matrix.^10–16^ The final properties are primarily governed by the filler-to-polymer weight ratio.^17,18^ This approach inherently forces a compromise: increasing filler loading to improve electrical conductivity often leads to increased stiffness and brittleness, while prioritizing mechanical compliance usually sacrifices conductive pathways.^19,20^ Furthermore, these bulk mixing methods are inherently limited in spatial resolution,^21^ making them unsuitable for fabricating miniaturized devices that require precise microscale patterning of different material properties.

Direct laser writing (DLW) has emerged as a powerful tool for high-resolution additive manufacturing.^22–24^ In the context of composites and hybrid materials, many DLW techniques have operated in a modification processing mode.^25–29^ This often involves writing within a predeposited, homogeneous film of composite material (e.g., a photoresist mixed with metal NPs),^30^ where the laser primarily alters local chemistry^31–33^ or structure^34^ but does not additively introduce new material phases with high spatial selectivity. A paradigm shift toward an additive processing mode, where distinct functional phases are deposited directly and selectively from a feedstock, could offer unprecedented control over the composition and properties of local materials. Such an additive processing mode has already been achieved in the context of direct laser metallization approaches. In laser-induced chemical liquid phase deposition (LCLD) and laser-induced thermal voxels (LITV), substrates immersed in a metal precursor are exposed to a focused laser and the local heating by absorption of laser thermally drives the redox process and produces metallic or oxide structures, but the deposition is mainly pure metal or oxides and the spatial resolution does not fully utilize the optical resolution.^35–38^ Besides the thermal effect, the wavelength of the laser can be tuned to the absorption band of metal precursors to excite the supramolecular complexes and induce the redox intramolecular reaction.^39,40^ Although it can produce metal/carbon composite nanoparticles of 20–30 nm size, the elemental composition ratio is predefined by precursor supramolecular complexes.

In this work, we explore a reagent-engineering strategy based on light-induced material deposition.^41,42^ The patterned deposition process relies on the concurrent action of two laser-induced processes at the focal point: the laser triggers the reductive photodeposition of metal or metal oxides on semiconductor NP surfaces,^43,44^ while the optical trapping force drives the assembly and bonding of these NPs onto the substrate. We hypothesize that this process can be functionally extended: if the chemical groups responsible for reduction are attached to a functional NP, then that NP would be incorporated into the growing deposit, allowing its intrinsic properties to directly tune the composite’s characteristics.^45,46^ Here, we demonstrate this principle by formulating a precursor that mixes a metal salt solution with a suspension of carboxylated red fluorescent nanodiamond (ND) from Adamas Nanotechnology. These red fluorescent nanodiamonds have a 3.5 ppm concentration of negatively charged nitrogen vacancy (NV) centers. When the NV center is illuminated by the green 532 nm laser, free electrons in the conduction band of nanodiamond are generated via a two-photon ionization process.^47,48^ The free electrons and the carboxylate group as a sacrificial agent contribute to the reductive photodeposition of metal or metal oxide.^49^ As the ionization rate scales quadratically with the excitation laser power, observable deposition of metal (oxide) and ND composite in our setup is achieved with irradiance over 300 mW/μm^2^. The steps and schematics of the reaction are illustrated in Figure 1.

**Figure 1:**
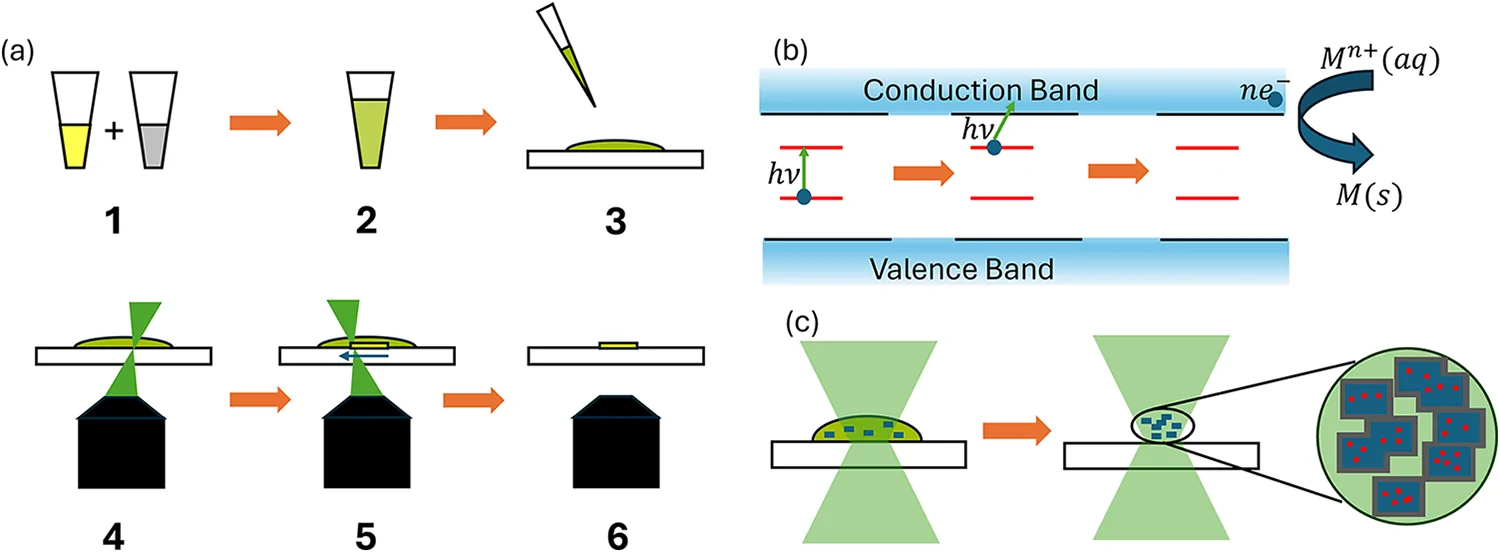
(a) Steps of laser-induced material deposition of metal (oxide) and nanoparticle composite patterns (1: metal precursor in yellow and nanoparticle suspension in gray; 2: reagent mixture of the two components; 3: the mixture is transferred onto the substrate by a pipet; 4: laser focused at the starting point of a pattern; 5: laser scanning to form a pattern; 6: residual mixture is rinsed away by water and the patterned composite deposition is on the substrate). (b) Microscopic view of the deposition process (from left to right): NV center is excited by a photon, then an electron in the excited state is excited by a second photon into the conduction band, and finally the electron in the conduction band triggers a reductive photodeposition. (c) Macroscopic view of the deposition process (from left to right): initially, the NDs are uniformly dispersed in the mixture, then the trapping force induced by the focused laser beam concentrates the NDs at the focus spot, and the photon flux (green background) and NV centers (red dots) trigger the deposition of metal (oxide) (gray outlines).

In this work, we show that this reagent-engineering strategy enables the direct laser writing of homogeneous metal/oxide NP composites. We demonstrate that varying the metal precursor tunes the electrical conductivity of the deposit over 6 orders of magnitude, while changing the size of the ND suspension modulates its mechanical hardness over an order of magnitude. Crucially, these two property axes can be controlled independently, as one is linked to the metal precursors in the solution and the other is linked to the NPs in the suspension. This approach establishes a pathway for the additive, high-resolution fabrication of composite materials with decoupled and spatially programmed functional properties.

## Results and Discussion

### Fabrication and Characterization of Nanocomposites

We developed a library of precursor solutions with different metal salts (H_2_PtCl_6_, FeCl_3_) and ND suspensions (Adamas Nanotechnologies, carboxylated ND (1 μg mL^–1^)). The ND suspension is sonicated for 5 min to ensure uniform distribution of ND particles. Then, the ND suspension is mixed with the metal precursor in a 1:1 volume ratio. The mixture is sonicated for another 5 min. The deposition process and the exposure parameters are listed in the Experimental Section.

To characterize the morphology and elemental composition of the composites formed by our reagent-engineering strategy, line structures of platinum (Pt) and Iron oxide are deposited on glass substrates from mixtures containing the respective metal precursor and ND particles. The morphology and elemental distribution of these deposits are characterized using scanning electron microscopy (SEM) coupled with energy-dispersive X-ray spectroscopy (EDS) (JSM-6390 JEOL).^50^ As shown in Figure 2a,b, SEM images (top row) reveal the formation of continuous lines for two composites (Pt + 100 nm NDs and iron oxide + 100 nm NDs), and Figure 3 presents the zoomed-in SEM image of the surface of 8 different composites (Pt + carbon ink, 60, 100, 140 nm NDs and iron oxide + carbon ink, 60, 100, 140 nm NDs). There are no observable cracks or strong aggregation of nanoparticle clusters on the surface of composites. The relatively smooth surface of the line pattern indicates the absence of a thermally driven process during deposition.^51–53^ EDS elemental mapping is subsequently performed on these lines. The maps for the primary metal signals (Pt, Fe in the upper left of each SEM image) and the carbon (C in the upper right of each SEM image) demonstrate a spatially coincident and uniform distribution across the deposited structures. The consistent overlap of metal and carbon signals indicates a homogeneous composite formation where ND particles are integrated within the metal matrix.

**Figure 2:**
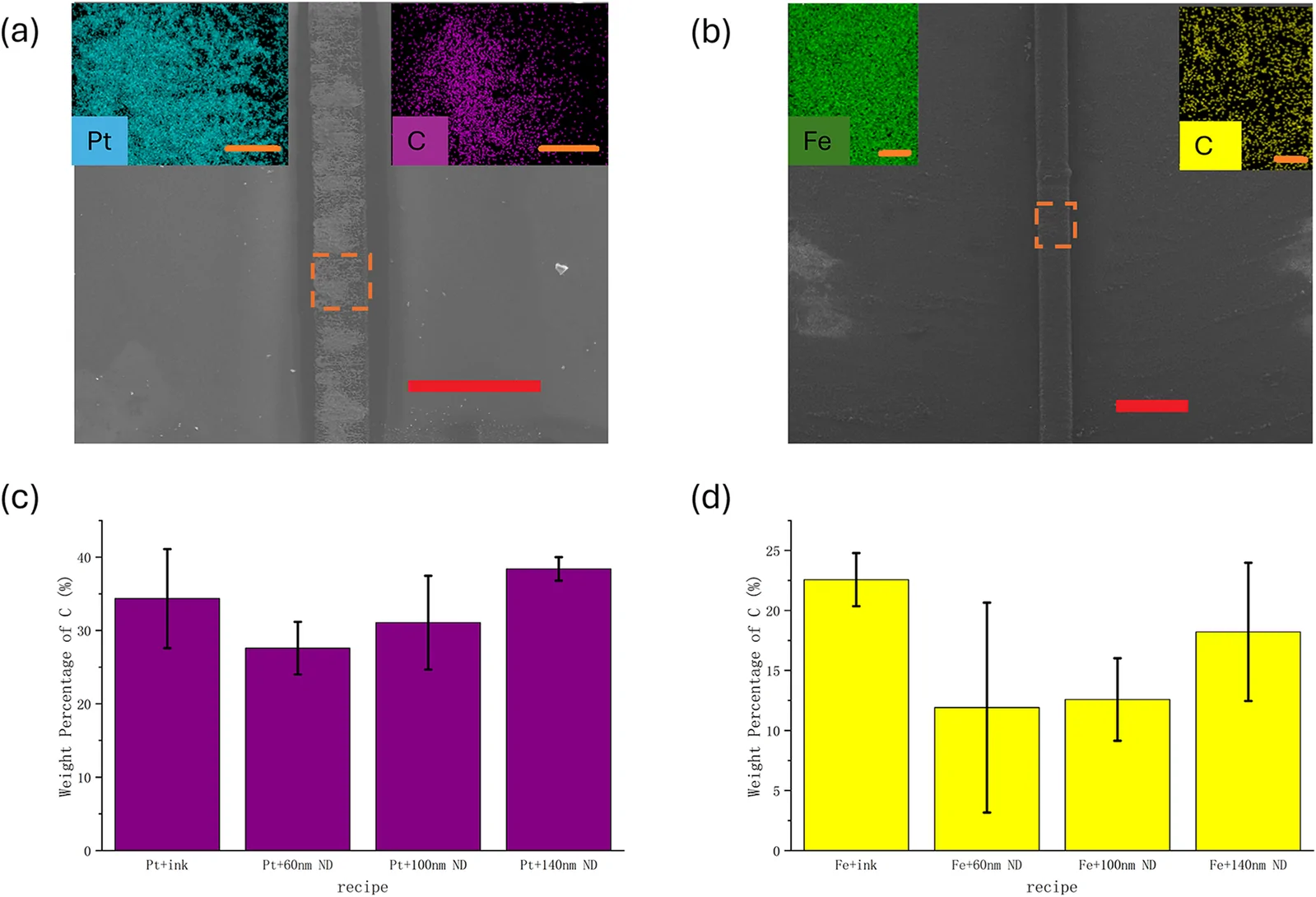
(a, b) SEM images of platinum (Pt) and iron oxide line deposition on a glass slide, respectively. The insetted images are EDS mappings of the corresponding elements in the region enclosed by the orange dashed line. The red scale bar in the SEM image is 100 μm and the orange scale bar in the EDS mapping (inset) is 10 μm. (c, d) Weight percentage of carbon measured by EDS in composite deposition using different NP suspensions.

**Figure 3:**
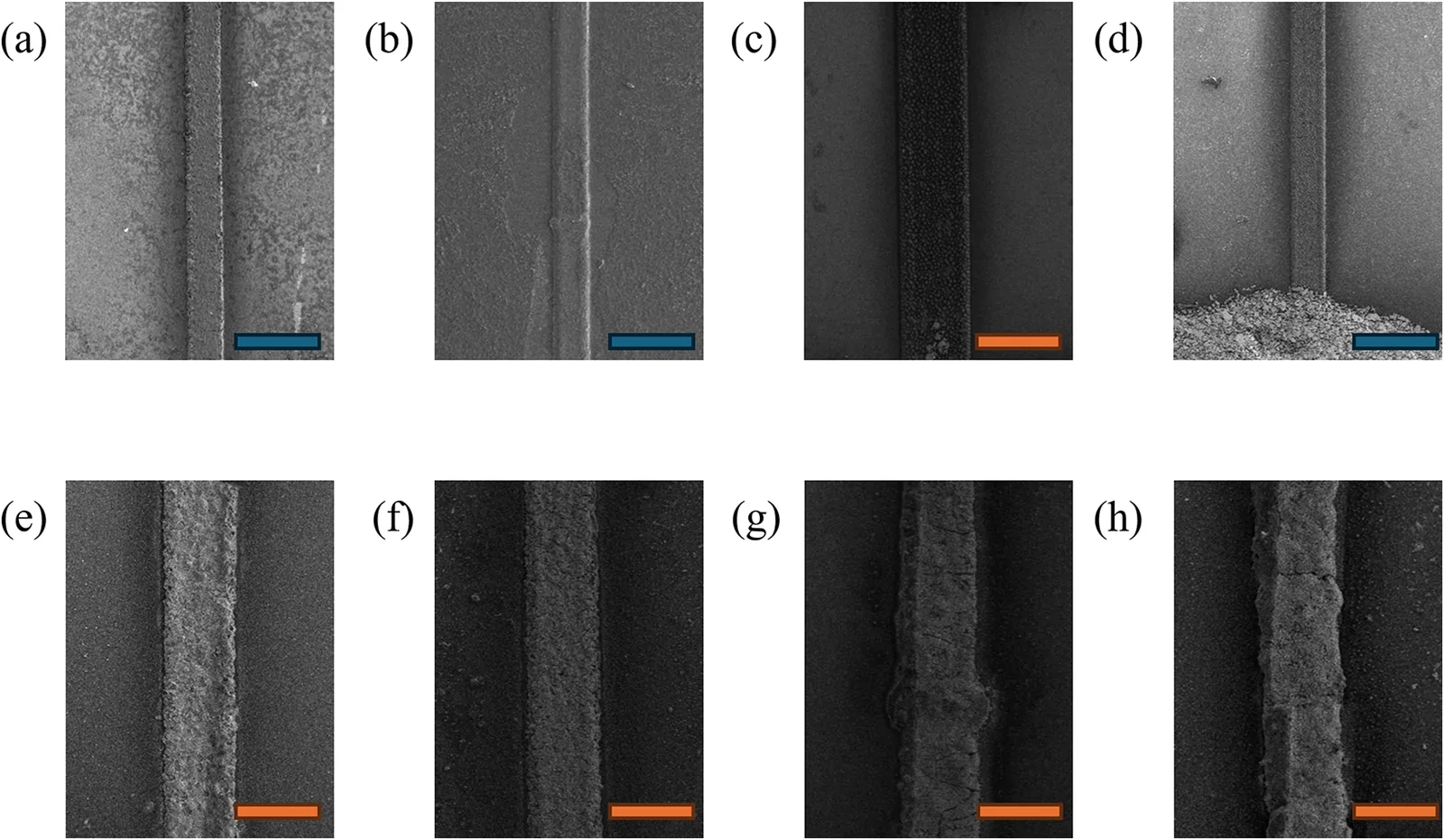
(a–d) SEM images of the surface of iron oxide composite deposition in combination with carbon ink, 60 nm ND, 100 nm ND, and 140 nm ND. (e–h) SEM images of the surface of Pt composite deposition in combination with carbon ink, 60 nm ND, 100 nm ND, and 140 nm ND. The blue scale bar is 100 μm and the orange scale bar is 50 μm.

To further characterize the structure of composites, a focused ion beam (FEI Helios G4 UX) is used to cut out the cross section of composites in Figure 4. Regardless of mixture composition, all of the composites are closely attached to the glass substrate with micrometer-scale thickness, and few holes are observed at the cross section. This homogeneity is fundamental to achieving consistent functional performance,^54^ as it ensures that the locally engineered properties dictated by the precursor composition, namely, the NP fraction for mechanical tuning and the metal/oxides for electrical tuning, are reliably manifested throughout the structure, forming a solid foundation for the property-programmable platform.

**Figure 4:**
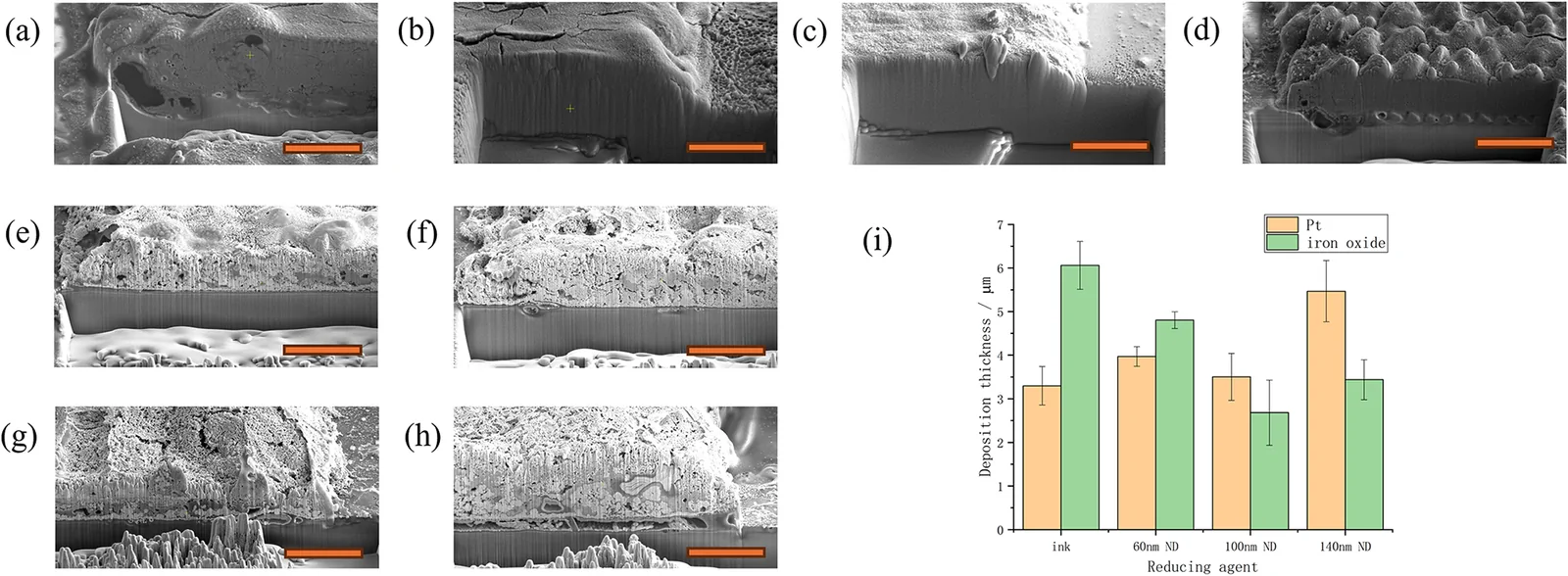
(a–d) SEM images of the cross section of iron oxide composite deposition in combination with carbon ink, 60 nm ND, 100 nm ND, and 140 nm ND. (e–h) SEM images of the cross section of Pt composite deposition in combination with carbon ink, 60 nm ND, 100 nm ND, and 140 nm ND. The orange scale bar is 5 μm. (i) The average thickness of Pt and iron oxide deposition reduced by different nanoparticles.

### Electrical Property of Nanocomposites

With the homogeneous microstructure established, we next quantified the functional properties of the composites and demonstrated their independent tunability. The electronic and mechanical responses are systematically characterized as functions of the two primary reagent components: the metal salt solution and NP suspension.

The electronic transport properties of the composites are systematically characterized to quantify the effect of the reagent composition by measuring their *I*–*V* curve and electrical conductivity. First, a series of line structures are fabricated on glass substrates using precursor solutions with different metal salts (H_2_PtCl_6_, FeCl_3_), but the same ND filler size (100 nm). Subsequently, the current–voltage (*I*–*V*) characteristics of each line are measured using a probe station, and the results are summarized in Figure 5a–c. The linearity of the *I*–*V* curve demonstrates that the contact between composite deposition and silver glue (H20E EPO-TEK) is ohmic.^55^ Since the composites follow Ohm’s Law, composites made from the same metal salt (H_2_PtCl_6_, FeCl_3_), but different NPs (carbon ink, 60, 100, and 140 nm ND), are prepared in a line structure for conductivity measurement. The cross-sectional dimensions (width and height) of the selected lines are measured by scanning electron microscopy (SEM) and focused ion beam (FIB) milling (FEI Helios G4 UX Dualbeam). The calculated conductivity values are summarized in Figure 5c, which reveals a decisive trend: varying the metal salt precursor alters the conductivity of the composite by several orders of magnitude from around 3000 S cm^–1^ for Pt to around 0.02 S cm^–1^ for Iron oxide. In contrast, modifying the size of the ND filler or changing it to carbon ink induces only a moderate variation in the conductivity. The consistency of electrical conductivity is more obvious in the case of Pt compared with iron oxide, as the conductivity of the latter is also affected by the extent of oxidation during deposition. Diamond is an insulator while the carbon ink is slightly conductive, but the electrical conductivity of Pt/ND and Pt/carbon ink composites are both about 1% of the bulk Pt. The decoupling between electrical conductivity in Figure 5d and weight percentage of carbon in Figure 2c,d demonstrates that the conductive pathway is defined almost exclusively by the percolating metal network formed from the metal salt precursor, establishing it as the primary variable for programming electronic properties in this composite system.

**Figure 5:**
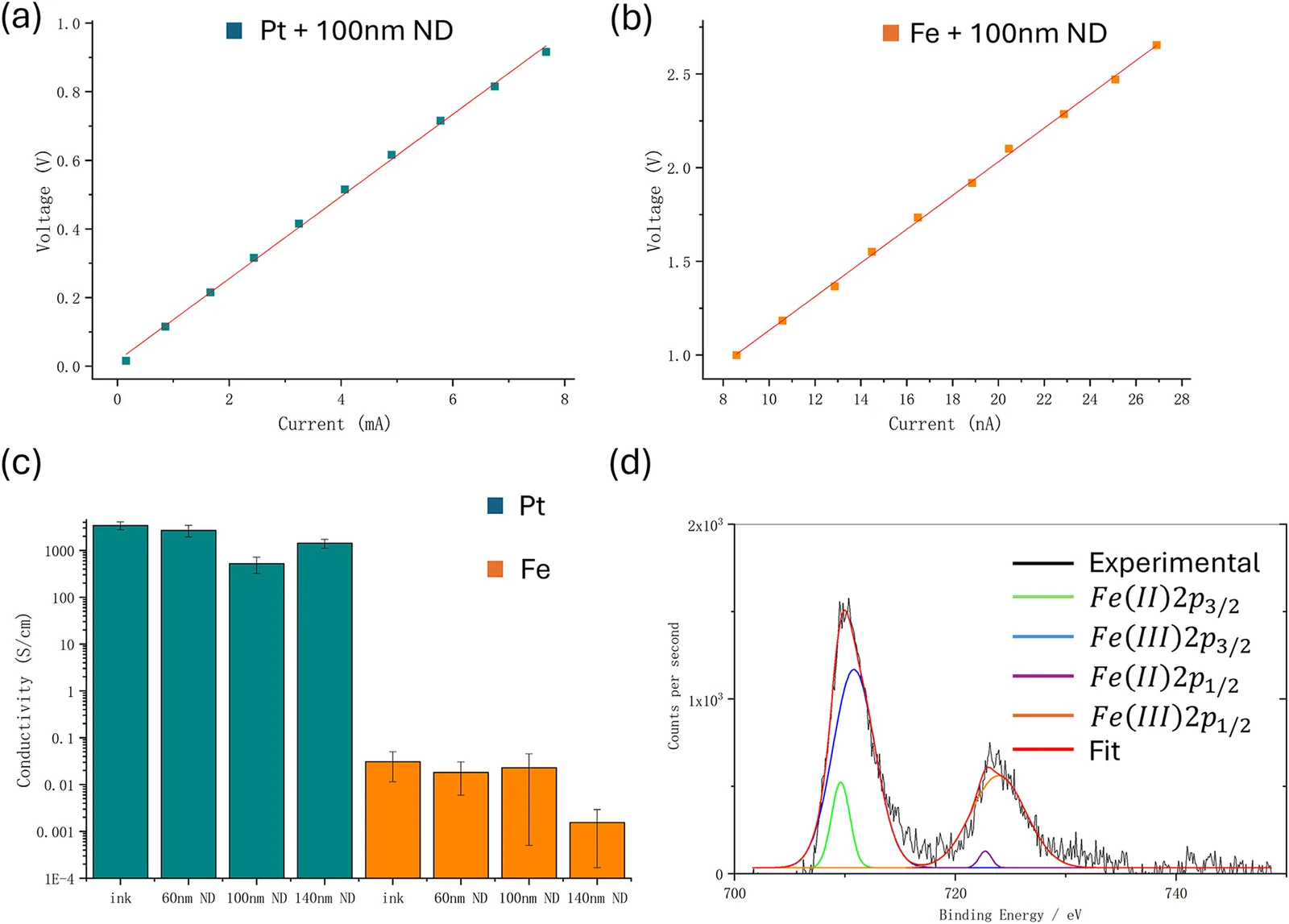
(a, b) *I*–*V* curves of Pt and iron oxide lines mixed with 100 nm ND, respectively. (c) Electrical conductivity of platinum and iron oxide lines mixed with ND of different sizes and carbon ink. (d) XPS of iron oxide lines mixed with 100 nm ND shows strong Fe oxide peaks and absence of Fe metal peaks.

### Mechanical Property of Nanocomposites

The mechanical properties of the composite, evaluated via nanoindentation, present a more complex relationship with the filler than do the electrical properties. A series of square pads are fabricated on glass substrates using precursor solutions with the same metal salts (H_2_PtCl_6_, FeCl_3_) but different sizes of NP filler (carbon ink, 60, 100, and 140 nm ND). The measured reduced elastic modulus *E* and hardness *H* of the composites are plotted as a function of ND size in Figure 6.

**Figure 6:**
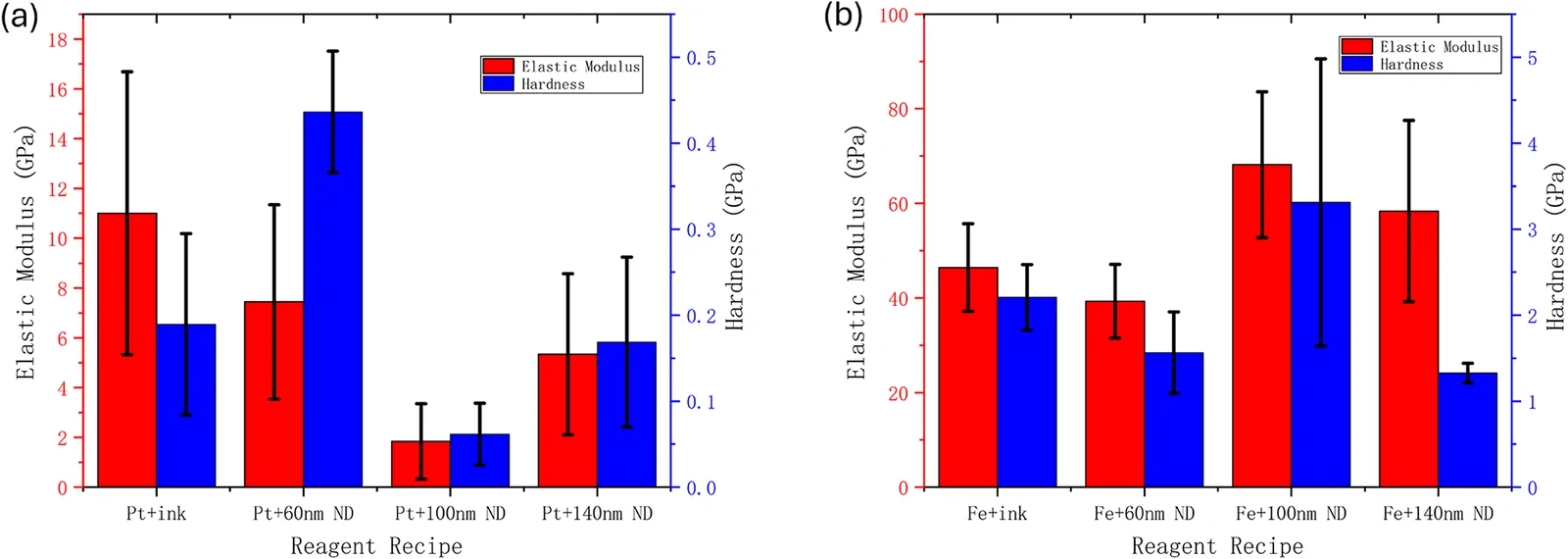
(a) Elastic modulus and hardness of platinum reduced by different NP suspensions. (b) Elastic modulus and hardness of iron oxide reduced by different NP suspensions.

The measurements indicate that both *E* and *H* are indeed influenced by the size of the ND filler. However, this influence does not follow a simple monotonic trend where larger particles invariably yield a higher elastic modulus or hardness. For instance, while platinum with 100 nm ND has the lowest elastic modulus of 1.84 GPa and hardness of 61.66 MPa, iron oxide with 100 nm ND has the highest elastic modulus of 68.2 GPa and hardness of 3.31 GPa.

This nonmonotonic behavior underscores that the mechanical parameters are not solely dependent on the chemical composition of the filler but are critically governed by the resulting composite microstructure. Factors such as the spatial distribution of NDs within the metal matrix, filler–matrix interfacial adhesion, and potential aggregation at certain size ratios could contribute to the final mechanical response.

### Property Map of Nanocomposites by Reagent Engineering

The functional capability of our reagent-engineered laser writing is summarized in a property map plotting electrical conductivity and hardness (Figure 7). The data points from our systematically varied precursor recipes form a broad quasi-rectangular envelope in the property space. It is a direct graphical signature of independent control: conductivity can be varied over orders of magnitude at nearly constant hardness (horizontal spread) by changing the metal salt precursor, while hardness can be tuned at fixed conductivity levels (vertical spread) by altering the NP suspension. This orthogonal tunability, achieved within a single fabrication step, demonstrates a key advantage of our direct laser writing approach for functional material design.

**Figure 7:**
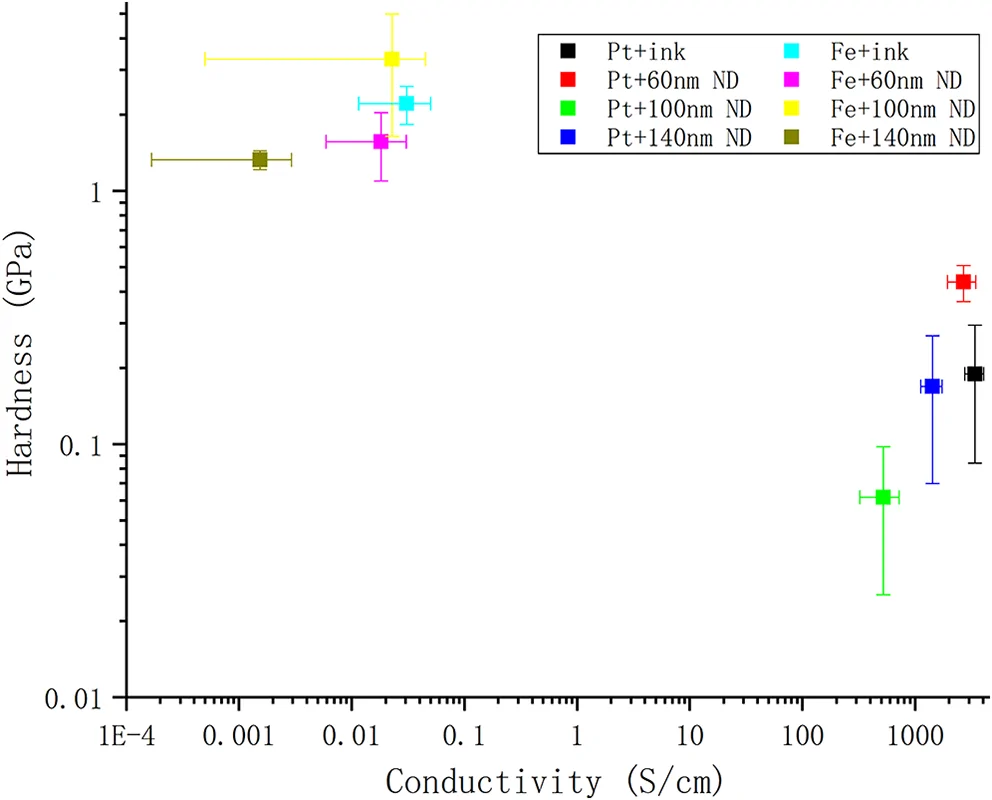
Property map of hardness and electrical conductivity of nanocomposites.

Furthermore, the range of conductivity and hardness accessed by our composites extends beyond the typical region reported for many conventional functional composites fabricated by bulk mixing or printing techniques, which often cluster along a narrow, coupled trajectory due to inherent property trade-offs.^56,57^ By decoupling the control mechanisms and expanding the accessible property space, our method provides a versatile platform for fabricating materials with customized functional performance that was previously difficult to achieve.

## Conclusions

In summary, we have developed a general reagent-engineering paradigm for direct laser writing that enables the decoupled design of functional composites with independently tunable electrical and mechanical properties. Using the model system of metal–ND composites, we demonstrate that orthogonal control over electrical conductivity (spanning 6 orders of magnitude) and hardness (tunable over 1 order of magnitude) can be achieved by separately programming the metal precursor and the NP suspension component within a single-step deposition process. The incorporation of NDs opens possibilities for functional devices. The NV centers in the embedded NDs can serve as spatially resolved quantum sensors for in situ strain and temperature mapping within the composite. With appropriate optical readout, these direct-laser-written composites could potentially enable multifunctional platforms where electrical conductivity, mechanical properties, and quantum sensing capabilities are integrated within a single microscale device.

Crucially, the design principle is not limited to ND fillers. As long as the NP is capable of generating free electrons from its band structure upon absorption of photons to reduce metal precursors, the NP and metal/oxide composite will interconnect with each other to form patterned deposition by laser scanning. In the case of photodeposition, it is the narrow band gap semiconductor that generates electron-hole pair, while in the case of wide band gap material like ND, it is color center defect that generates free electrons through an ionization process. The methodology establishes a versatile platform where the NP identity, whether ceramic, polymeric, or carbon-based, can be selected to tailor specific mechanical, thermal,^58–60^ or optical responses,^61,62^ while the metal salt independently defines the electronic characteristics. By decoupling these property axes and occupying a broad, unprecedented region on the material-property map, this work transcends conventional trade-offs in composite fabrication. It offers a scalable, in situ compatible strategy for creating functionally graded architectures, opening pathways toward next-generation devices in flexible electronics,^63^ biointegrated sensors, and adaptive microsystems that require spatially programmed performance.

## Experimental Section

### Chemicals and Precursor Preparation

All of the metal precursors used in this work are commercially available and of analytical grade. The ND suspension is purchased from Adamas Nanotechnologies with a concentration of 1 mg mL^–1^ dispersed in deionized water. The carbon ink is purchased from Japanese Kaguro Ink. All metal precursor solutions are prepared using deionized water as the solvent. The metal precursor is obtained by dissolving metal salts/metalate in deionized water at 30 mmol L^–1^ for hexachloroplatinic­(IV) acid hexahydrate (H_2_PtCl_6_·6H_2_O) and 50 mmol L^–1^ for iron­(III) chloride (FeCl_3_). Separately, a colloidal suspension of NDs (Adamas Nanotechnologies, Carboxylated Red Fluorescent ND with average sizes of 60, 100, and 140 nm) is sonicated for 5 min and diluted in deionized water to 1 μg mL^–1^. The carbon ink is diluted in deionized water to 1/1500 of its as-purchased concentration. The metal salt solution, carbon ink, and the ND suspension are individually sonicated for 5 min to ensure homogeneity. Subsequently, these two components are mixed at a 1:1 volume ratio and stored in centrifuge tubes. Every time before using the mixture for deposition, the mixture is sonicated for 5 min to ensure homogeneity. The final concentrations of metal precursor and nanoparticles in the respective reagent mixture are 15 mmol L^–1^ for H_2_PtCl_6_·6H_2_O, 25 mmol L^–1^ for FeCl_3_, 0.5 μg mL^–1^ for NDs, and 3000 times dilution of carbon ink.

For every round of deposition, only 30 μL of the mixture is transferred onto the substrate. To monitor the stability of the mixture, UV–vis absorption spectroscopy (Lambda 1050+ (PerkinElmer)) is performed, and the spectra of mixtures when freshly prepared and stored in the dark for 5 days are compared in Figure 8.^64^ From the spectra, the mixture of metal precursor and ND suspension does not degrade over the examined period. In addition, the weak absorption at 532 nm excludes the potential of thermally driven reduction of the metal precursor. The absorption of 532 nm photons due to the two-photon ionization process is not observable in the spectra because the concentration of carbon in the nanodiamond suspension is only 0.083 mmol L^–1^, and the concentration of NV centers and surface carbon atoms connected to the carboxylate group is about 6 orders of magnitude lower than that.

**Figure 8:**
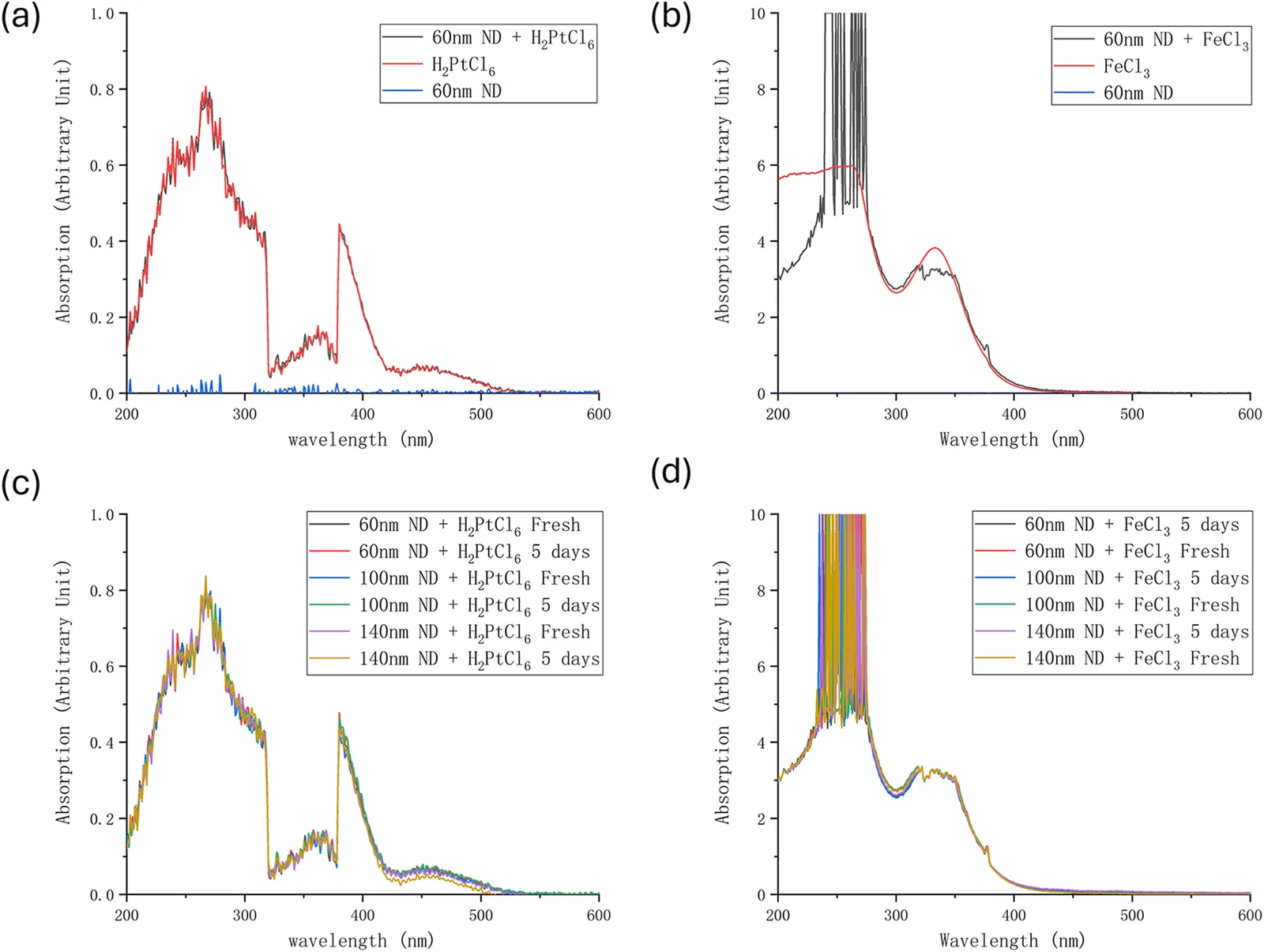
(a) UV–vis absorption spectra of 60 nm NDs, H_2_PtCl_6_, and their mixture. (b) UV–vis absorption spectra of 60 nm NDs, FeCl_3_, and their mixture. (c, d) UV–vis absorption spectra of metal precursor and ND mixture when freshly prepared and stored in the dark for 5 days.

### Direct Laser Writing Setup and Exposure Parameters

The direct laser writing setup is shown in Figure 9.^41^ A continuous-wave laser source (1) operating at a wavelength of 532 nm (Laser Quantum) is directed by a mirror (2) and a 532 nm notch filter (5) into a set of galvo mirrors (Sino Galvo) (6). The laser beam scanning is realized by a 4f scanning system formed by the galvo mirrors (6) and two plano-convex lenses (7) with a 200 mm focal length. The laser beam is then deflected upward by another mirror (2) and passes through the back aperture of an objective (Nikon 20×/0.75 air) (8). The glass slide (10) is mounted on an XYZ manual stage (9). The mixture (11) is transferred to the substrate by a pipet. The laser is focused at the mixture–substrate interface. The mixture–substrate interface is simultaneously illuminated by a white LED (12) from above. And the transmission optical image passes through (following the black dashed line) the 532 nm notch filter and is focused by a lens (4) of 100 mm focal length on a CCD camera (3) for monitoring the deposition process. Writing is performed by scanning the laser focus at a constant speed of 45 μm s^–1^ across the substrate surface.

**Figure 9:**
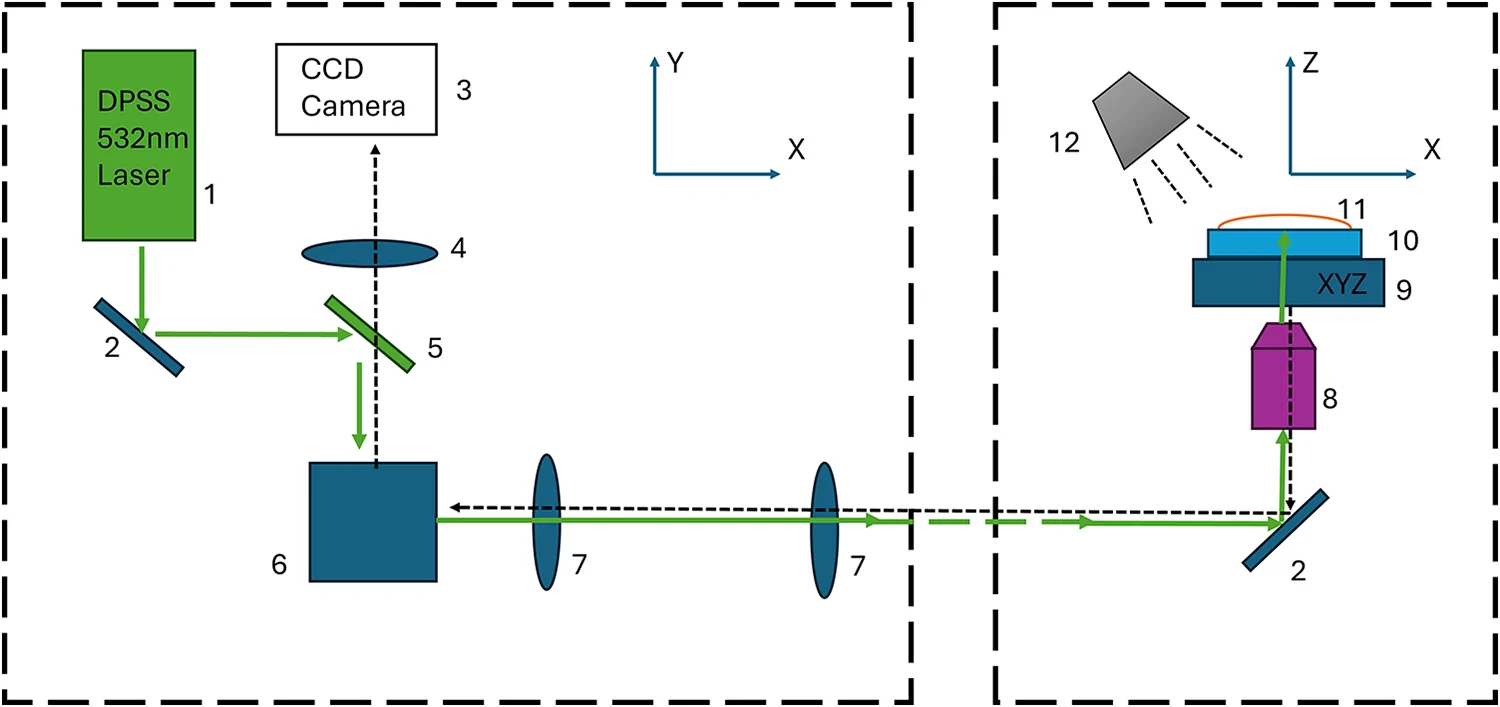
Block scheme of the experimental setup for laser-induced deposition of Pt composites and iron oxide composites.

The on and off state of the laser is controlled by an Acousto-Optic Modulator (AOM) (G&H AOMO 3350-199). The laser power for different mixtures is determined by first increasing the laser power until a deposition is observed on the CCD camera instantaneously when the AOM is switched on, then starting the laser scanning and further increasing the laser power until continuous composite deposition is observed for all mixtures using the same metal precursor. The laser power is measured after the second lens (7) in the 4f system and before the deflective mirror (2). The laser power is 57.6 mW (irradiance at 582 mW/μm^2^) for mixtures containing H_2_PtCl_6_ and 30 mW (irradiance at 303 mW/μm^2^) for mixtures containing FeCl_3_. The samples for SEM imaging, FIB nanofabrication, EDS measurement, and electrical measurement are 50 μm × 800 μm lines of composites produced from the respective mixtures. And the samples for the nanoindentation experiment are 50 μm × 50 μm squares of composites produced from the respective mixtures. All depositions are conducted under ambient conditions on cleaned glass substrates (GLASWARENFABRIK KARL HECHT).

### Characterization of Electrical Property and Mechanical Property

The electrical resistivity of the deposited composite lines is determined by combining the resistance and cross-sectional area measurements. First, conductive silver glue (H20E EPO-TEK) electrodes are applied to both ends of the line shape composite deposition on a glass substrate. Current–voltage (*I*–*V*) characteristics are measured using a probe station with a source measure unit (Keithley 2400). To calculate electrical conductivity, the resistance is derived from the linear Ohmic region of the *I*–*V* curve. The cross-sectional area of the line is measured by imaging a cross-section cut made using a focused ion beam (FEI Helios G4 UX Dualbeam) with a scanning electron microscope shown in Figure 4. The cross sections of different composite depositions are consistently solid and thick. Young’s modulus and hardness of deposition are extracted from the loading and unloading curves measured by nanoindentation (Hysitron, Triboindenter TI 900). The indentation depth was 750 nm for Pt deposition and 200 nm for iron oxide deposition.

## Supplementary Material













## Data Availability

Data for this article, including SEM images, *I–V* curves, and loading and unloading curves of composite deposition, are available at DataSpace@HKUST at https://dataspace.hkust.edu.hk/dataverse/limdfunctional.

## Notes

Data for loading and unloading curves of composites (ZIP)
EDS spectrum and mapping of composite materials (ZIP)
Fe dimension (ZIP)
Fe IV curve (ZIP)
Nickel IV curve (ZIP)
Pt IV curve (ZIP)
